# Searching for qualitative research for inclusion in systematic reviews: a structured methodological review

**DOI:** 10.1186/s13643-016-0249-x

**Published:** 2016-05-04

**Authors:** Andrew Booth

**Affiliations:** Reader in Evidence Based Information Practice, School of Health and Related Research (ScHARR), University of Sheffield, Regent Court, 30 Regent Street, Sheffield, S1 4DA UK

**Keywords:** Qualitative research, Systematic reviews, Literature searching

## Abstract

**Background:**

Qualitative systematic reviews or qualitative evidence syntheses (QES) are increasingly recognised as a way to enhance the value of systematic reviews (SRs) of clinical trials. They can explain the mechanisms by which interventions, evaluated within trials, might achieve their effect. They can investigate differences in effects between different population groups. They can identify which outcomes are most important to patients, carers, health professionals and other stakeholders. QES can explore the impact of acceptance, feasibility, meaningfulness and implementation-related factors within a real world setting and thus contribute to the design and further refinement of future interventions. To produce valid, reliable and meaningful QES requires systematic identification of relevant qualitative evidence. Although the methodologies of QES, including methods for information retrieval, are well-documented, little empirical evidence exists to inform their conduct and reporting.

**Methods:**

This structured methodological overview examines papers on searching for qualitative research identified from the Cochrane Qualitative and Implementation Methods Group Methodology Register and from citation searches of 15 key papers.

**Results:**

A single reviewer reviewed 1299 references. Papers reporting methodological guidance, use of innovative methodologies or empirical studies of retrieval methods were categorised under eight topical headings: overviews and methodological guidance, sampling, sources, structured questions, search procedures, search strategies and filters, supplementary strategies and standards.

**Conclusions:**

This structured overview presents a contemporaneous view of information retrieval for qualitative research and identifies a future research agenda. This review concludes that poor empirical evidence underpins current information practice in information retrieval of qualitative research. A trend towards improved transparency of search methods and further evaluation of key search procedures offers the prospect of rapid development of search methods.

## Background

The contribution of qualitative evidence to health care decision-making is increasingly acknowledged. Qualitative evidence syntheses (QES) now occupy an important role within the activities of international collaborations, such as the Cochrane Collaboration [1], as part of the guidance production processes of national organisations such as the UK National Institute for Health and Clinical Excellence (NICE) and the US Agency for Healthcare Research and Quality (AHRQ) and as a genuine academic endeavour funded by private, public and charitable funding bodies. Increasingly, QES are viewed as a putative mechanism by which the systematic review “catechism” can be advanced from “what works” to “what happens” [2]. Milestones for the development of QES methodology are well-documented [3]. They include the publication of the first methodology for qualitative synthesis (meta-ethnography) in 1988 [4], the formal recognition of the Cochrane Collaboration’s Qualitative Methods Group in 2006 and publication of the first Cochrane QES in 2013 [5].

Data compiled for the annual Evidence Synthesis of Qualitative Research in Europe (ESQUIRE) workshop in 2015 suggests that between 40 and 70 qualitative syntheses are published each month across a wide range of disciplines with 2–5 methodological references on qualitative synthesis appearing within the same period.

The most cited QES methodological guidance is Chapter 20 of the Cochrane Handbook, authored by co-convenors of the Cochrane Qualitative Methods Group [6]. This was the first document to recognise the potentially important role of qualitative research within the Collaboration. Space constraints limited the searching section to three paragraphs which covered the usefulness of filters, the importance of supplementary searching strategies, an early attempt to highlight the importance of sampling decisions and a cursory sentence on reporting standards. Following receipt of a methodology grant and subsequent methodological summit in Adelaide, the Cochrane Qualitative Methods Group produced supplementary guidance hosted on the Group’s Website. Chapter 3 of this supplementary guidance covered searching for studies [7] mirroring the trial-focused chapter on searching from the Cochrane Handbook. Then, Cochrane policies confined qualitative research to a supporting role within collaboration activities [3] which resulted in potentially useful guidance on supplementary approaches to searching being relegated to an Appendix.

At about this time, the Centre for Reviews and Dissemination was revising its guidance on conducting systematic reviews. For the first time, a chapter on qualitative systematic reviews was included in this seminal guidance. Chapter 6 entitled “Incorporating qualitative evidence in or alongside effectiveness reviews” consisted of 20 pages including just over two pages related to identification of the evidence [8]. Topics covered included a characterisation of search procedures, single paragraphs on sampling approaches and supplementary strategies, respectively, a lengthy discussion of search strategies and filters and a single sentence on reporting standards.

Despite considerable advances in QES methodology, many gaps remain to be addressed. While this is true for all stages of the review process, the place of searching at the beginning of the process renders it a particular priority. Our knowledge of searching for qualitative research is founded primarily on custom and practice. Very few empirical studies exist to inform information retrieval practice. Consequently, we have an imperfect knowledge of the most effective retrieval terms, partial understanding of the respective yield of different sources and, in particular, an incomplete insight of the appropriateness of different sampling methods as they relate to different types of QES.

This methodological review was compiled to support the work of the author and other co-convenors of the Cochrane Qualitative and Implementation Methods Group in writing updated guidance on literature searching for qualitative evidence. In conjunction with a pending major revision of the Group’s chapter in the Cochrane Handbook, the co-convenors of the Group have developed a publishing plan for supplementary guidance, including a chapter on searching. This methodological overview does not duplicate the forthcoming guidance. It documents the evidence base that will inform the guidance, much as a systematic review might inform subsequent clinical guidelines. The aim is to produce a summary of the evidence base for searching for QES that is not constrained by current interpretations of the role of QES within the Cochrane Collaboration. Such a methodological summary may conceivably inform handbooks and other guidance as produced by health technology assessment (HTA) agencies, guideline producers and other review organisations.

Specifically, the author sought to address three methodological questions:

- What is the current state of knowledge in relation to this aspect of searching practice?
- How robust is the evidence base for this aspect of searching practice?
- What are the main gaps and future research priorities for this aspect of searching practice?

## Methods

Systematic approaches to searching may be typified by seven characteristics (“the 7S structure”). As highlighted in a recent paper, systematic retrieval requires a transparent method for producing a structured review question, the availability of search strategies (or filters) to assist in sifting relevant studies from those likely to be irrelevant, and replicable and evidence-based search procedures that can be enhanced and adapted to each particular review [9]. An additional challenge is associated with choosing which sources to search and learning the idiosyncrasies of each source [10]. These requirements extend to three further characteristics—standards for reporting search strategies, an informed strategy for sampling studies and judicious use of supplementary search methods. These seven characteristics were used to structure findings from the review, following a summary of existing overviews and guidance for information retrieval in QES.

Typically, a methodological review requires the conduct of sensitive searches across multiple databases. However, for the last 7 years, the author has maintained the study register of the Cochrane Qualitative and Implementation Methods Group. This is populated on a monthly basis by sensitive keyword searches of PubMed MEDLINE (Appendix) and the ISI Web of Science and by citation searches for key methodological books and journals on ISI Web of Science and Google Scholar. Reference lists from new articles and book chapters are checked regularly for further additions to the register. The register is the single most comprehensive database source of (i) methodological references on all aspects of QES and (ii) published examples of QES. A sensitive search was therefore conducted of the study register on Reference Manager 12 using such terms as “search*,” “retriev*” and “database*.” Six hundred fifty-four references were retrieved, and these were reviewed for relevance by the author. A high proportion of references were anticipated to be “false hits” as they reported the search methods used for specific QES. However, this sensitive search strategy increased confidence that all relevant methodological sources would be retrieved.

Previous guidance on searching for qualitative evidence was used to compile a list of 15 key citation pearls on various aspects of retrieval. A citation pearl is an authoritative article, typically identified by experts, of particular relevance to the topic of inquiry that can be used to search for relevant and authoritative materials sharing common characteristics with the original pearl [11]. Searches on Google Scholar using each title in quotation marks were used to identify all references citing these pearls (i.e. as indicated with “Cited By”). Including duplicates, 1063 references were identified from these 15 citation pearls (Table 1). Therefore, 1717 references were identified through the two search approaches. Once duplicates were removed, 1299 records were available.

**Table 1:** Fifteen citation pearls in literature searching for qualitative research

Reference	No. of citations	Category
Barroso et al. (2003) [10]	152	Overviews
Booth (2006) [12]	92	Standards
Cooke et al. (2012) [13]	46	Question formulation
Evans (2002) [14]	116	Overviews
Finfgeld‐Connett and Johnson (2013) [15]	21	Overviews
Flemming and Briggs (2007) [16]	90	Filters
Grant (2004) [17]	35	Filters
Grayson and Gomersall (2003) [18]	59	Sources
McKibbon et al. (2006) [19]	45	Filters
Papaioannou et al. (2010) [20]	54	Supplementary strategies
Shaw et al. (2004) [21]	146	Filters
Subirana et al. (2005) [22]	31	Sources
Walters et al. (2006) [23]	31	Filters
Wilczynski et al. (2007) [24]	48	Filters
Wong et al. (2004) [25]	97	Filters
Total	1063	

Relevant references were coded under one or more of eight headings used to structure this review, namely Overviews and Methodological Guidance, Sampling, Sources, Structured Questions, Search Procedures, Search Strategies and Filters, Supplementary Strategies and Standards. Searching, sifting and coding were conducted in July/August 2015 [10, 12–25].

The author examined the full-texts of all items identified for inclusion from the searches, most having been previously assembled to support the methodological work of the Cochrane Qualitative and Implementation Methods Group. Two types of evidence were used in compiling this structured methodological review. Empirical studies were examined to assess their practical implications for those conducting QES. Methodological commentaries, overviews and guidance handbooks were inspected in order to construct a snapshot of current practice.

Data to answer the three review questions was extracted into a single spreadsheet using Google Forms. The 7S framework was used as a structure for data extraction. Narrative text extracts from each article were cut and pasted into the data extraction framework. In addition, papers were categorised for study design and empirical studies examined for the quality of their design. A narrative commentary was produced to summarise both the included references and findings from the extracted data.

## Results

A total of 113 items were identified for inclusion in the methodological review [10, 12–120]. Of these, 46 were characterised as overviews of QES methodology, 13 represented formal guidance on conduct or reporting with a further 3 being narrative reviews specifically of the QES search process. One paper was a short general summary and could thus not be classified as an overview in the literal sense. Seven papers used a hybrid design that combined an overview with one (*n* = 4) or multiple (*n* = 2) case studies and, in one case, with both a survey and a case study. The remaining 43 papers employed a formal study design and are described more fully below.

**Fig. 1:**
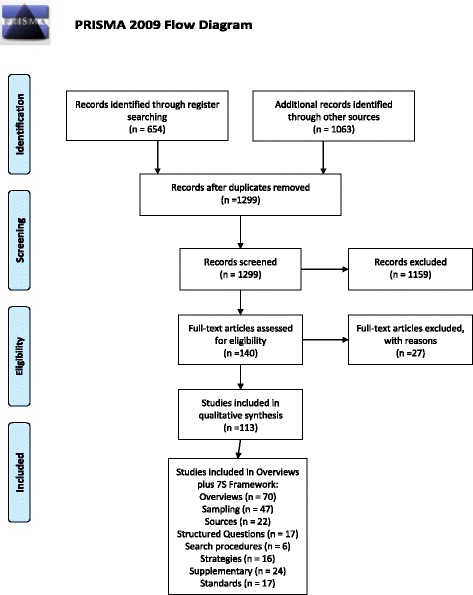
PRISMA 2009 flow diagram

With regard to the seven components of the 7S framework, the papers were distributed as follows: Sampling (*n* = 47), Sources (*n* = 22), Structured Questions (*n* = 17), Search procedures (*n* = 6), Search Strategies and Filters (*n* = 16), Supplementary Strategies (*n* = 24), and Standards (*n* = 17). The aggregate number of papers exceeded 131 items indicating that some papers, particularly overviews, contributed to more than one of the 7S components (Fig. 1—flow chart). Although conclusions based simply on “vote-counting” should be resisted, it is noteworthy that issues of sampling are frequently discussed, in marked contrast to a default of comprehensive sampling typically used when conducting quantitative systematic reviews. In comparison with quantitative systematic reviews, there was also greater emphasis on the use of a variety of sources and of supplementary search methods.

With regard to the quality of the included papers, it was not possible to perform quality assessment for the body of included papers because (i) many papers were narrative offerings (*n* = 68) and (ii) even where a formal study design (*n* = 43) was present, the heterogeneity of these designs and the lack of a common appraisal instrument made comparability across studies prohibitively challenging. Observations on the robustness of the evidence base for each aspect of searching practice were therefore constrained to types of supporting designs. Specific observations are included within each of the following component sections. However, overall, the studies can be characterised as follows: case study (25); comparative study (7); literature surveys (5); multiple case studies (1); surveys (1); and validation study (4). Unsurprisingly, case studies of individual QES were the most common investigative design with a single paper reporting multiple case studies within the same paper. Five literature surveys identified a set of eligible studies and then examined reporting of methods within the study set. One study surveyed those who conduct literature searches. In terms of more robust designs, seven studies used a comparative design and four studies investigating the use of search filters attempted validation against a gold standard.

Many authors document the perceived complexity of searching for qualitative research, either in comparison with quantitative research (particularly randomised controlled trials) [8, 14, 36, 39] or in its own right [10, 25]. As a consequence, searching for, and identifying, appropriate qualitative research is characterised as “frustrating and difficult” [22]. Table 2 summarises the most frequently documented challenges.

**Table 2:** Challenges when searching for qualitative research studies

Limitations
Variation of use of the term “qualitative” [8, 17]
Variety of qualitative methodologies (e.g. ethnography, phenomenology and grounded theory) [44]
Non-standardised terminology for qualitative research [44]
Use of descriptive non-explicit titles [8, 14, 30, 34, 37, 40, 45]
Variable content and quality of abstracts [8, 14, 37]
Lack of structured abstracts [8, 14]
Absence of abstracts [8, 34, 40]
Absence of research method from abstracts [14, 37]
Absence of clear descriptions of study samples in the published abstracts [47]
Inadequacy of indexing terminology for qualitative methodology [8, 14, 21, 26, 30, 33, 34, 37, 40, 44]
Inappropriate assignment of index terms by indexers [8, 21, 46]
Inter-database differences in indexing terminology [8, 14, 30, 31, 37]
Potential mismatch between focus of paper and focus of the review [34]
Non-existence of registers of qualitative research [8, 26, 34]
Qualitative research located outside medical databases [43, 45]
Absence of pointers to qualitative research from registers of RCTs [8]
Difficulty in identifying qualitative reports associated with RCTs [6]
Difficulty in retrieving reports of mixed-methods studies [6]
Social science employs more diverse publication media than medical literature [18, 37]
Strategies for qualitative research can be over inclusive, time-consuming and expensive [21, 46]

While some challenges are being addressed, for example with the addition of the Medical Subject Heading (MeSH) term to MEDLINE in “Qualitative Research” in 2003 [34] and with the trend towards more informative titles and abstracts, progress appears slower than in the more researched domain of quantitative research. Established methods for identifying quantitative research do not necessarily translate into effective strategies for qualitative research [34]. Dixon-Woods et al. [30] reported that 23 % of records screened for a qualitative review of support for breastfeeding did not include an abstract. As a consequence, a higher proportion of full-text articles may need to be screened to make decisions about inclusion [45].

### Overviews, summaries and guidance

For inclusion in this section, a publication (i.e. peer-reviewed book chapter, journal article or report) was required to either (i) provide an overview of the entire literature searching process, or substantive components of this process, in the specific context of qualitative research or (ii) attempt a methodological overview or analysis of one or more methods of qualitative synthesis including a consideration of literature searching methods. Forty-six items were overviews of QES methodology [14, 15, 18, 26–28, 30–32, 35, 36, 38, 43, 47–51, 53–55, 57–59, 61, 63, 64, 66, 67, 69, 70, 75–79, 83, 85, 86, 89, 91, 98, 99, 102, 103, 113], and further seven overviews were combined with one [10, 29, 41, 52] or multiple case studies [37, 40] and, in one instance, with both a survey and a case study [46]. Thirteen papers represented guidance on conduct or reporting [6–8, 33, 34] [80, 88, 97, 100, 117–120], three were narrative reviews of the QES search process only [106, 111, 112] and there was one general summary [71].

These overview texts reflect a range of approaches to literature searching. Some simply translate the comprehensive model for quantitative studies to a qualitative context [47]. However, increasingly, overviews acknowledge differences between aggregative and configurative reviews [48] and the respective merits of comprehensive and purposive sampling [49].

### Sampling

For inclusion in this section, a publication was required to either (i) provide an overview of sampling in the context of QES or (ii) include a substantive discussion of the topic of sampling within a published synthesis, or (iii) mention sampling within works identified for the “Overviews, summaries and guidance” section. Forty-seven items were thus included in this section [4, 6, 8, 10, 12, 30, 32, 34, 35, 37, 38, 40, 42, 45–47, 49–66, 68–76, 78–80, 82].

While there is general agreement on the need for search strategies to be systematic and explicit, recent debate focuses on whether QES share the need for comprehensive, exhaustive searches [69]. Some argue that a more purposive sampling approach, aiming to provide a holistic interpretation of a phenomenon, where the extent of searching is driven by the need to reach theoretical saturation rather than to identify all eligible studies [6, 30, 34, 46], might be more appropriate. Systematic reviews of qualitative research inhabit the point at which two research traditions meet [38]. On the one hand, the methodology of systematic reviews, developed principally over the last two decades, has been dominated by quantitative systematic reviews. Systematic reviews (SRs) of trials attempt to locate every possible study on a given topic or intervention [35]. Some authors [10, 63] advocate a similar approach for QES. On the other hand, Booth [54] argues that, rather than adopting a “trials-type search,” authors should use a “theory” driven approach, resembling “diversity” or “saturation” sampling approaches used in primary qualitative studies. The interpretive nature of QES suggests the value of methods derived from primary qualitative research, such as the use of theoretical sampling until data saturation is reached [54]. Whereas in quantitative meta-analysis, omission of a key paper is critical to statistically drawn conclusions; this is not true of a QES which aims to make a conceptual and interpretative contribution. Campbell et al. affirm that “omission of some papers is unlikely to have a dramatic effect on the results” [37]. The creative tension that results from bringing together these two traditions, labelled as a “dual heritage” [38], is most clearly seen in the literature that describes how to select an appropriate sample.

Selection of an appropriate sample of participants is fundamental to quantitative and qualitative research [121]. Similarly, selection of an appropriate sample of papers is essential for a successful evidence synthesis [38]. In quantitative reviews, this is typically framed in terms of a “comprehensive” sample, by implication a universal sample, to minimise bias and to permit subsequent generalisation. Conceptually, however, a comprehensive sample is problematic as it constrained by the number and type of resources to be searched, the diversity of materials contained within such sources and the time available. Recently, I proposed that “exhaustive” be preferred over “comprehensive” because it conveys the finite nature of resources (e.g. searcher time, money, and access to databases; time to sift). “Exhaustive” is relative to the purpose and type of review rather than referencing some mythical absolute [76]. However, such debate is clouded by the tendency to use “comprehensive” and “exhaustive” interchangeably. Brunton et al. [76] observe that “exhaustive searching is improbable,…the obligation on reviewers is to plan a thoughtful and clearly described plan [sic] to locate the sample of studies most likely to answer their research question[s] reliably.” Key difficulties include how to establish the population of potentially relevant studies without identifying all relevant studies [45]. Data or theoretical “saturation” could have limitations in this context; importantly, how can a reviewer know that an additional study will not add important insights? [46].

Early commentators expressed anxiety that selective sampling may result in the omission of relevant data, thus limiting understanding of the phenomenon [50, 53, 60]. Gallacher et al. [40] characterise two schools: those who advocate using purposeful sampling to retrieve materials until data saturation is reached [51] and those who aim to retrieve all relevant studies in a field rather than a sample of them [8]. The first approach has logistic and epistemological drivers and is often taken when review teams face a large and diverse set of resources [61] or when they are developing concepts and theories [65].

Studies aimed at comprehensively summarising the literature include a comprehensive and rigorous search using predefined index/subject heading/free-text terms, informed by an initial scoping search [10, 30, 64]. Thus, aggregative reviews, characterised by the Joanna Briggs Institute’s proprietorial meta-aggregation method, explicitly seek to mirror the breadth of sources included within an effectiveness review [49]. Such an approach facilitates comparability between quantitative and qualitative outputs. However, it pays scant recognition to the different epistemological traditions underpinning different types of qualitative synthesis.

For a qualitative reviewer, time is best spent not “piling up examples of the same finding, but in identifying studies that contain new conceptualisations of the phenomena of interest” [76]. Notwithstanding good methodological justifications for searching comprehensively in SRs of trials, not least to protect against systematic errors such as publication bias [76, 80], qualitative data collection is characterised, not by “statistical representativeness” but by “systematic non-probabilistic sampling” [122]. Several authors [52, 54, 55, 65] suggest drawing on the sampling techniques of primary qualitative research, including theoretical sampling and theoretical saturation, when synthesising qualitative literature. Booth [54] states that the intention of QES is not to identify all literature on a particular topic, the aim being identification of papers with characteristics relevant to the phenomenon being studied, not statistical representativeness [54]. Innovative techniques might be “borrowed” from primary qualitative research such as deliberately seeking studies to act as negative cases, aiming for maximum variability and designing results set to be heterogeneous, as an alternative to “the homogeneity that is often the aim in statistical meta-analyses” [32].

Downe [71] describes how theoretical saturation might be operationalised in terms of whether additional studies continue to reinforce the line of argument. Under such circumstances, the author reasons, a search for new studies will reap increasingly diminutive returns, offering justification for truncating the search. O’Connell and Downe [73] describe how they identified a point of theoretical saturation “when two articles identified late in the search process did not add anything new to the emerging synthesis.” One reviewer reflects that their background as a quantitative systematic reviewer pushed them towards a higher threshold: “I support the ‘data saturation approach’ and think if the next twenty papers don’t offer anything new, what’s the likelihood of the 21^st^ (reflexive statement)” [46]. A more mechanical, rather than interpretive, interpretation of saturation relates to the recurrence of *studies*, rather than *themes* [63], as references to the same study begin to reappear repeatedly.

The benchmark of a comprehensive sampling frame persists despite the methodological innovation offered by purposive and theoretical sampling approaches. Adding additional electronic databases to a search protocol (i.e. to search for more of the same) [22] runs counter to the strategy of seeking to diversify a sample (i.e. purposively to move on to different, more productive lines of inquiry). Several authors comment on the value of identifying the disconfirming case [30, 38, 54, 71], and search strategies may be targeted specifically to achieve such insights [38].

A reconciled position would state that the sampling method should be appropriate to the type of review and its purpose. The Centre for Reviews and Dissemination (CRD) guidance recognises the absence of consensus over the issue of sampling [8]. Rather than predicate their guidance on epistemological concerns, they suggest that if the number of studies is too large to work through, researchers may decide to adopt a strategy for limiting the number of included studies. Purposive and/or theoretical sampling are the main choices, with papers selected for inclusion on the basis of such criteria as rich description or conceptual clarity [8]. The guidance highlights a role for random sampling, probably most appropriate when constructing a test set for a methodological investigation. Cited examples include purposive sampling derived from qualitative meta-synthesis [58] and critical interpretive synthesis [65].

Reconceptualising literature searching for QES, and indeed knowledge synthesis more generally, around the appropriateness of the sample rather than its completeness opens up an exciting variety of sampling approaches derived from qualitative research [78, 79]. Patton’s 16 strategies [123] could ultimately be matched to the full range of synthesis types in an expanded version of Table 3 and then translated into corresponding search techniques for each sampling method.

**Table 3:** Synthesis methods with appropriate sampling methods

Synthesis method	Description	Sampling method	Rationale for sampling method
Critical interpretive synthesis	A method of synthesis that offers a means of systematically producing explanatory theories directly from the data.	(1) Purposive sampling; (2) Theoretical sampling [65]	(1) Purposive sampling of representative cases used to immerse team in area of investigation. (2) Followed up by pursuit of further lines of theoretical inquiry.
Grounded theory-based approaches	An interpretive approach to synthesis that is modelled on the primary research methods of grounded theory.	Theoretical sampling [34]	Further lines of inquiry and hence routes for searching emerge from ongoing analysis of the data and hence require follow up along lines suggested by theory.
Meta-aggregation	A structured, process-driven approach to systematic review of qualitative research modelled on the conventional systematic review of quantitative literature as practised by the Cochrane and Campbell Collaboration.	Comprehensive sampling [49, 68]	Seeks to identify all relevant studies in order to establish credibility in conventional systematic review terms.
Meta-ethnography	An interpretive method for synthesising qualitative research of particular value in developing models that interpret findings across multiple studies.	Purposive sampling [56, 57] Theoretical sampling [34]	Interpretive focus places premium on identifying studies to contribute added value over and above current version of synthesis and thus requires sampling on a theoretical basis.
Meta-interpretation	A meta-synthetic approach used specifically in interpretative synthesis.	Maximal divergent sampling/maximum variation sampling [70, 72]; Theoretical sampling [70]	Focus on interpretation requires that insights are maximised by exploring papers that are not characteristic of the “average sample.”
Meta-narrative synthesis	Takes paradigmatic approach to map literatures from different research traditions.	Purposive sampling of key papers [35, 62]	Seeks an illuminative sample of papers from within different research traditions.
Qualitative meta-synthesis	Attempts to integrate results from multiple different but inter-related qualitative studies with interpretive, rather than aggregating, intent, in contrast to meta-analysis of quantitative studies.	Comprehensive (representative) sampling [55]	Patterned on conventional systematic review methods therefore seeks all relevant studies to represent entire phenomenon of interest.
Realist synthesis	Approach to complex social interventions or programmes which provides explanatory analysis aimed at discerning what works for whom, in what circumstances, in what respects and how.	At different points uses variously: (a) Comprehensive sampling [74]; (b) Purposive sampling [66]; (c) Theoretical sampling [34]; (d) Snowball sampling [61]	Comprehensive sampling (a) used to explore key focus of review. Becomes starting point for more explanatory exploration (b–d) of associated literature and mechanisms.
Scoping review	Rapid review that aims to map existing literature in a field of interest in terms of volume, nature, and characteristics of primary research.	Random sampling [74]	Aims to characterise literature, not to document studies in minute detail, sampling representative body of literature may suffice for planning purposes.

#### How many studies are enough?

A further cause for debate relates to the number of studies to include within a QES [34, 71]. Some methods of synthesis, such as meta-aggregation and meta-study, make a virtue of being able to handle large numbers of studies. More interpretive approaches privilege smaller numbers of studies [12, 71]. While it is undesirable to talk in terms of specific numbers [124], the amount of relevant data may be a function both of the number of studies and their conceptual richness and contextual thickness [8]. Furthermore, data considered rich and thick in relation to one aspect of the review question may be scarce in relation to another aspect, even within the same set of studies. Too few studies may limit the support for the entire synthesis or for individual constituent themes. Too many included studies may impair the data analysis, making conceptual analysis “unwieldy” or making it difficult to maintain insight or “sufficient familiarity,” [37] thereby obscuring patterns that are apparent within a smaller set of studies [46, 59, 68]. In seeking such a balance, we typically arrive at a preferred number of between 6 and 14 studies. Campbell et al. [37] suggest that “a maximum of about 40 papers is realistic because it is difficult to maintain sufficient familiarity with >40 papers when trying to synthesise them all….” Where the number of studies to be included falls short of the total population of eligible studies, for whatever reason, it becomes critical that “reviewers explicitly and transparently state their criteria for including studies” [34].

#### When can I stop searching?

In qualitative research, analysis and data collection occur simultaneously, often to the point where no new ideas are developing [46]. Thus, it is unlikely that a reviewer can pre-specify a set number of studies without considering their richness, thickness and overall quality. For situations other than exhaustive sampling, a reviewer must develop clear explanations for the circumstances under which searching was terminated. Stopping rules have been proposed, initially for methodological reviews, but these may apply, by extension, to reviews that seek to achieve saturation [82]. Quantitative reviewers currently seek methods to define a point beyond which further literature searching has little justification [30]. For qualitative reviews, the answer may lie in the principles of data saturation [30].

#### Sampling issues

The use of alternatives to comprehensive searching potentially creates several problems, particularly where a reviewer has chosen to locate their QES within a systematic review paradigm [34]. Some commentators express concern that alternative sampling approaches open a QES to allegations of subjective decision-making [75] or assertions that such reviews are no longer transparent or reproducible [30, 34]. Others respond that “systematic” should not be misappropriated to favour one research system over an equally legitimate alternative [38]. Nevertheless, pressure to observe quantitative systematic review conventions persists with 81 % of published meta-ethnographies using exhaustive search strategies [42]—this for a methodology that, as seen in Table 3, recognises the appropriateness of purposive sampling approaches. Indeed, the originators of the meta-ethnographic approach caution against exhaustive inclusion of data as it is likely to lead to over-generalisation and “trite conclusions” [4].

Just as there is no consensus regarding the number of interviews required for a “good” qualitative study, there is no consensus on what type of sample is required for a good qualitative synthesis [46]. France et al. [42] identify a need for further exploration of those circumstances under which exhaustive searches are desirable or necessary. Pragmatically, review teams need to bear in mind, when sampling, the underlying theoretical perspective together with a need to be explicit about any strengths and weaknesses of their approach [71]. This includes a requirement to communicate their sampling approach, methods used and the rationale that underpins the sampling approach.

Finally, while epistemological distinctions may occasion different sampling strategies, this distinction may not be quite so apparent in searching practice. The need to search extensively and to follow up any potentially useful lines of inquiry, while not driven by statistical considerations, may be no less present when seeking to find qualitative studies [32].

### Sources

For inclusion in this section, a publication was required to either (i) provide an overview of sources to be used in the context of QES or (ii) include a substantive discussion of the topic of selection of sources within a published synthesis or (iii) mention the selection of sources within any of the works identified for the “Overviews, summaries and guidance” section. A total of 22 items was thus included in this section [6, 8, 14, 18, 22, 34, 37, 39, 45, 46, 63, 83–85, 87–94].

#### Coverage of databases

In a sample of QES published in 2012–2013, Wright et al. [94] reports the number of databases searched per review ranged from 3 to 20, with 37 % searching from 3–5 databases, 28 % searching from 6–8 databases, 14 % searching 9–11 databases and another 14 % searching 12 to 14 databases. Seven per cent of reviews searched over 16 databases. Reliance on MEDLINE alone is particularly discouraged [83]. However, a meta-ethnographic study in complementary and alternative medicine searched 67 different database sources and yet found 87 % of included qualitative studies from PubMed alone [90]. CINAHL and DIMDI (a German database) also yielded a high number of relevant hits.

Several authors point to the superiority of CINAHL’s coverage of qualitative research [6, 8, 14]. The Cochrane Handbook [6] and CRD [8] highlight that CINAHL introduced “qualitative studies” in 1988, reflecting a particular interest in qualitative studies for nursing researchers while MEDLINE did not add a corresponding subject heading until 15 years later. However, technical efficiency should not be confused with coverage with CINAHL at 4.5 million records covering approximately a quarter of the records included by MEDLINE. In their recent study of multiple QES, Wright et al. [94] demonstrate that, assuming a rigorous search strategy and accurate indexing, CINAHL is a good source of primary studies with the potential to yield unique studies.

Surveys consistently report MEDLINE and CINAHL as the two most frequently searched sources of qualitative research [39, 84]. CRD guidelines [8] stipulate that “assuming that the topic of interest falls within their scope, then searching both MEDLINE and CINAHL is likely to be important.” CINAHL and MEDLINE retrieved references most relevant to a search on nursing manpower and EMBASE did not provide substantive additional information [22]. They recommend that both CINAHL and MEDLINE be consulted when planning an optimal bibliographical search related to nursing topics as differences in coverage were striking.

While publication bias possesses a lower profile within qualitative than quantitative research, review authors must be aware that limiting a search to well-known databases may result in missing useful information. In particular, review teams should identify specialist databases that relate to a particular topic and databases that contain particular types of publication, e.g. Dissertation Abstracts and supplementary search strategies that may increase the chance of finding grey literature or of retrieving journals not indexed by the mainstream databases [34]. McGinn et al. [91] report the performance of databases across a small set of social care topics; CINAHL performed at a consistently moderate level of sensitivity across topics, and Social Care Online performed consistently poorly. Social Services Abstracts (SSA) was the best performing database [91] although this again is likely to be topic specific [125] with certain databases being indicated for certain kinds of questions [87]. Some databases favour organisational-type questions while others privilege more clinical-type questions. McGinn and colleagues [91] observe an “unpredictability” around database performance across topics. This occasions researchers to use conservative risk-averse strategies such as consulting greater numbers of databases and screening larger numbers of hits. As in the relatively well-developed area of health care, there is an ongoing need for database comparison case studies across a wider variety of subtopics, thereby building up a body of evidence on retrieval for qualitative research.

National bodies commissioning reviews typically seek strong representation of indigenous studies within the evidence base for a particular review question. Stansfield et al. [92] demonstrated that a UK-fortified set of seven additional databases (British Education Index, Child data, IBSS, Index of British Theses, Social Care Online, The British Library Integrated Catalogue and Zetoc) yielded additional unique studies. Importantly, they did not only limit themselves to examining retrieval rates but also attempted to assess the impact on findings from the final review. Of five studies identified through UK-fortified strategies, one study was central to development of a descriptive theme while the other four less influential studies added detail and strength to the review’s findings. Furthermore, these studies were of generally high quality, contrasting with the methodological “futility” encountered in a corresponding investigation of effectiveness studies [126]. This represents an important future direction for evaluation of search sources, strategies and procedures.

#### Grey literature sources

Grey (or “fugitive”) literature (e.g. technical reports, working papers) is frequently cited as an important source. Commonly, reviewers pay homage to the potential value of searching grey literature and then reject its feasibility, citing limitations of time and costs [22, 37, 91]. In actuality, we know little about the impact of publication bias specifically on qualitative research. We understand that researchers will often want to avoid opening up the prospect of time-consuming and minimally productive follow-up of the unpublished literature. Nevertheless, it is unhelpful for reviewers to imply through terse reporting that they have “taken care of” this uncontrollable mass of alternative publications. McGinn et al. [91] describe how they pragmatically accepted grey literature, identified serendipitously, when an item satisfied search selection criteria but not specifically searching for it. This may open up a review team to charges of being “unsystematic.” We consider it unhelpful either to plan to search the “grey literature” or to claim to have done so—it is preferable to pre-specify exactly what forms of literature are being sought and then to select sources and strategies for these specific forms, e.g. theses, process evaluations, hospital internal reports, research reports, conference proceedings, studies produced by charities. Some authors have compiled lists of grey literature sources specific to health care [85] and social care [87, 127].

#### Books and book chapters

Several commentators highlight that qualitative research is published in books as well as journal articles. Strategies for searching books and book chapters require particular consideration [34, 37, 45, 63]. Some differences relate to the social science disciplinary background being substantively different from the literature of medicine [18, 37]. Campbell et al. describe inclusion of books and book chapters in their two meta-ethnography case studies [37]. They identify the limitation of “truncation bias” in connection with journal articles as the full details of a descriptive qualitative study are unlikely to be published in a short article. Campbell et al. therefore recommend using multiple databases and search strategies in order to maximise the yield of relevant qualitative papers [37]. Searching for books may be achieved through relevant organisational websites, book catalogues, Google Scholar and consultation with librarians [88, 89]. The determining factor is likely to be the resources available to an individual project [37].

#### Theses

Further potentially useful sources, particularly given that a qualitative research project is typically feasible within the constraints of an academic qualification, are dissertations and theses [46]. Some authors exhibit a similar resistance to including theses as they do for grey literature in general. Stated reasons for this range from practical considerations, to keep the number of papers manageable [37, 46] and to prioritise the literature that is easier and quicker to access [37], through to concerns about items not being peer reviewed and published reports [46]. Searching for theses is challenging as they are not indexed in the same way as journal articles and may be accessible only from experts (researchers, providers, policy makers) or via specialist theses databases [37, 93]. Access to relevant studies may be achieved by searching relevant organisational websites, Google Scholar, thesis databases, specialist journals and consultation with librarians [88, 89]. It may be feasible to include only recent theses as they are less likely to possess published journal counterparts [37]. A unique methodological issue is the depth of reporting possible in a PhD thesis. If one or two theses are included alongside a larger number of published articles, constrained by word limits, they may “swamp” the data from these naturally thinner studies. It is preferable, where possible, to identify published journal articles derived from theses, thereby making the units of analysis more readily comparable. Nevertheless, the volume of data from more extensively reported theses is not an argument against their inclusion per se, simply against the uncritical use of theses, making procedures of quality assessment correspondingly more critical.

### Structured questions

To be included in this section, a publication should (i) provide an overview of structuring of review questions within the context of QES or (ii) include a substantive discussion of structuring review questions within a published synthesis or (iii) any relevant mention in works identified for the “Overviews, summaries and guidance” section. A total of 17 items was included in this section [9, 10, 13, 15, 28, 30, 31, 34, 41, 65, 73, 95–98, 101, 102].

The literature on qualitative searching tends to reflect four approaches to use of a structured, formulated review question. A minority of commentators assume that the requirement to formulate the question as a Population-Intervention-Comparison-Outcome (PICO) is shared across quantitative and qualitative review types [101]. The PICO format is underpinned by assumptions derived from epidemiological study design seen in the terminology used. A large majority of variants propose modifications that reinterpret the PICO approach, e.g. 3WH [28], PEICO(S), PICo, PICOC, PICOS, SPICE, SPIDER [98]. Several commentators suggest specific question formulations for specific purposes, e.g. CIMO [96], ECLIPSe [95] BeHEMoTh [9]. Noticeable among these second and third approaches are the typical addition of elements capturing context (aka environment or setting, e.g. BeHEMoTh, CIMO, PEICO(S), PICo, PICOC, SPICE) and the stance of the affected party (e.g., perspective in SPICE and stakeholders in PEICO(S)) (Table 4).

**Table 4:** Notations for qualitative question formulation

Notation	Components	Source
3WH	What (topical), Who (population), When (temporal), How (methodological)	[28]
BeHEMoTh	Behaviour, Health context, Exclusions, Models or Theories	[9]
CIMO	Context, Intervention, Mechanisms, Outcomes	[97]
ECLIPSe	Expectations (improvement, innovation or information), Client group (recipients of service), Location (where service is housed), Impact (what change in service and how measured), Professionals involved, Service	[95]
PEICO(S)	Person, Environment, Intervention, Comparison, Outcomes, (Stakeholders)	[98]
PICO	Patient/Population, Intervention, Comparison, Outcomes	[139]
PICo	Population, phenomenon of Interest, Context	[102]
PICOC	Patient/Population, Intervention, Comparison, Outcomes, Context	[31]
PICOS	Patient/Population, Intervention, Comparison, Outcomes, Study type	[101]
SPICE	Setting, Perspective, Intervention/phenomenon of Interest, Comparison, Evaluation	[96]
SPIDER	Sample, Phenomenon of Interest, Design, Evaluation, Research type	[13]

A significant few question the appropriateness of a pre-specified (i.e. a priori) question at all. Drawing upon the primary qualitative heritage of grounded theory, they assert that the review question only emerges from a preliminary analysis of the data. Related to this issue are two other considerations; first, whether iterative approaches are appropriate to searching for qualitative research and second, when a QES accompanies an SR of trials, whether the scope of the review questions should be coterminous.

As with quantitative reviews, there is little empirical data to support the merits of question formulation [13, 101]. With regard to the choice of specific frameworks, limited evidence suggests that PICO may be preferred when the primary objective is sensitivity whereas SPIDER favours specificity [101]. The authors recommend a modified PICO with added qualitative search terms (PICOS) which optimises the trade-off between sensitivity (not missing relevant items) and specificity (only retrieving relevant items) for circumstances when a fully comprehensive search is not feasible.

#### Differences between quantitative and qualitative review questions

Lorenc et al. [41] describe circumstances where the qualitative review question may not directly mirror the PICO of an SR of trials. In their review of preventive interventions for skin cancer, they identified useful non-interventional studies that were about attitudes to sun behaviours or skin cancer in general [41]. They reinterpreted their inclusion criteria to include any study reporting qualitative evidence relating to sun protection beliefs or behaviours, regardless of a link to a specific intervention. They conclude that this finding might translate to public health, and social and health research more generally, where relatively little qualitative evidence on specific interventions is available. Data linked to specific interventions were not necessarily of greater value than data related to broader attitudes.

Where QES are conducted in parallel with SRs of trials, review teams may have to adopt different conceptual schemata for their inclusion criteria and search strategies [41]. In contrast to current guidance, that seeks a common question structure (e.g. PICO) for the SR of trials and the QES, the authors flag that “structural divergence” between the two questions may be inevitable [41].

O’Connell and Downe [73] attempt to reconcile the tension between the need to preserve flexibility and yet “maximise rigour” through an explicit two-stage process. This process involved iteration in regard to topic definition followed by tight control over inclusion and exclusion, study quality and analysis. Scoping a topic, primarily performed for logistic considerations in an SR of trials, becomes correspondingly more important if the review team is to ensure “secure” concepts within the context of a QES. Divergence between commentators on the need for a pre-specified formulated question is partly explained by whether they consider the scoping process to be preliminary to, or integral to, the review process. So Ring et al. [34] state that QES typically start with a relatively well-defined research question and yet acknowledge, with Dixon-Woods et al. [30] that, according to their philosophical approach, some QES reviewers modify their initial research question in response to literature searching and screening [34].

Some commentators make a useful distinction between summative or aggregative QES, where research questions are generally established a priori and relevant research reports are identified exhaustively, and knowledge-building or theory-generating QES where such pre-specification may inhibit creativity [15]. The latter, they argue, starts from a less-clearly defined focus and evolve iteratively [10]. Within such a context, the expansive, as opposed to exhaustive, literature search can be viewed as a creative vehicle for continually redefining the research question and exploring the emergence of research findings. Consequently, Dixon-Woods and colleagues [65] evoke earlier qualitative researchers in suggesting that this process, with the review question being highly iterative and modified in response to search results, treats the question as a compass rather than an anchor.

### Search procedures

Publications in this section should either (i) provide an overview of topic-based database search procedures within the context of QES or (ii) include a substantive discussion of topic-based database search procedures within a published synthesis or (iii) mention database search procedures within works identified for the “Overviews, summaries and guidance” section. Six items were thus included in this section [15, 21, 34, 35, 37, 103].

A literature searcher faces a dual challenge in how best to optimise the trade-off between recall and precision, thereby keeping the expenditure of resources within manageable limits [37]. Several variables determine appropriate search procedures. These include how diffuse or broad the topic for review or synthesis is which requires a wider net and inclusion of more databases [37]. Exhaustive searches often necessitate “trawling” to identify every possible study. They often prove time-consuming and result in large numbers of non-relevant studies [34]. Strategies that attempt to maximise the number of potentially relevant records (high sensitivity) often result in a large number of non-relevant studies (low specificity) [21]. A review team should seek to optimise the ratio between the number of relevant references and the number of retrieved references (the “hit rate”) for sensitive topic-based searches and reflect whether available time might be better spent conducting citation searches on the Web of Science or Google Scholar (see the “Supplementary strategies” section). Finfgeld-Connett and Johnson [15] distinguish between extensive search approaches (that map to exhaustive searches) and expansive approaches (which progressively explore emerging lines of inquiry).

Mackay [103] differentiates between “qualitative” and “quantitative” searching approaches highlighting a similarity to the difference between qualitative and quantitative research methods. A quantitative searching approach is linear and structured based on objective and reproducible identification of pre-specified literature. Qualitative search approaches are concerned with the essential and peculiar character of phenomena and recognise that searching is never value free. Qualitative searching strategies are slow, labour intensive and difficult to replicate (because of the amount of time needed). They may be used when a topic is not dominant in the discourse of the literature and/or the topic is not well-conceptualised in the literature. The comprehensive a priori quantitative search contrasts with the intuitive and recursive follow-up of the purposive, iterative qualitative search. Both strategies can be systematic or not depending on how disciplined the searcher is. Documentation of the a priori (protocol-driven) search strategy (e.g. using screen captures) ensures that the search is explicit and thorough. For the iterative searching approach, a process analogous to memoing may be used to record the working notes of the searcher. Thus, both qualitative (iterative) and quantitative (a priori) search approaches can be systematic if the searcher is explicit about their searching processes.

Even though all synthesis methods include iteration, the degree, and the review stage at which iteration takes place, varies. Framework synthesis and critical interpretive synthesis explicitly involve iterative literature searching while realist synthesis and meta-narrative involve iteration at every stage [35]. Several synthesis methods do not explicitly mention iterative searching and thus implicitly subject themselves to a priori and positivist assumptions [35]. Meta-aggregation follows closely the single pass a priori*-*formulated search strategy model, the “big bang approach” which relies upon pre-identification of searching strategies, inclusion/exclusion criteria etcetera and implementing these with fidelity [35]. Increasing awareness of the array of sampling methods available and appropriate for synthesis coupled with the pragmatic demands of conducting reviews in public health or social work practice is likely to result in wider uptake of iterative searching. However, iterative searching poses significant challenges to the reporting of search strategies and may well subvert the discipline imposed by the Preferred Reporting Items for Systematic Reviews and Meta-Analyses (PRISMA) flow diagram [128].

The process of development of a search strategy for a QES is not demonstrably different from that for an SR of trials [34]. In both cases, searches need to be developed for the topic area and, separately, for the types of studies to be included [34]. The searcher must judge the optimal balance between sensitivity (not missing relevant items) and specificity (only retrieving relevant items) for both the topic and the study type. A very specific interpretation of qualitative research might involve only searching for words relating to ethnographies. A sensitive interpretation might involve specifying the types of phenomenon (e.g. views, attitudes, feelings), the study types (e.g. phenomenology), the data collection methods (e.g. interviews, questionnaires and focus groups) and the types of data (stories, narratives, etcetera).

A related issue concerns the type of data source to be included within the QES. The Cochrane Qualitative and Implementation Methods Group concurs with other review teams (e.g. [10, 28, 37]) by operationalising “qualitative research” as research using a recognised method of qualitative data collection and a recognised method of qualitative data analysis. More forgiving interpretations might include data from open-ended responses to questions in an otherwise quantitative questionnaire or survey. A narrow set of studies might be retrieved by only synthesising qualitative data reported in or alongside randomised controlled trials (e.g. from pilot studies, feasibility studies, process evaluations). We coined the concept “sibling studies” to characterise studies that derive from the same parent study but that report a particular slice of the data [7, 110]. Glenton et al. [104] explored the use of directly related (sibling) qualitative studies in connection with a Cochrane review of lay health workers. Only a small proportion of included trials had carried out some form of qualitative data collection during or after the intervention. Data were “sparse” with methods and results being poorly described. Their findings echo an earlier study by members of the same team [105] that found only 30 of 100 trials had associated qualitative work. Furthermore, around half of these sibling studies pre-dated publication of the trial [104].

Reviewers may also decide either to include or exclude mixed-methods studies and may choose either to synthesise such studies in their entirety or to focus only on the qualitative component of the larger study [8]. However, CRD guidance cautions against relying on strategies designed to retrieve clinical trials as a route to identifying qualitative associated or linked counterparts, citing the Cochrane Handbook requirement for structured searching [129].

The scope of an SR of clinical trials may differ from that for an accompanying QES [41]. Qualitative researchers may not have conducted research around a particular intervention, particularly where it is novel or experimental. A review team may need to access research about the patient’s experience of their condition, barriers and facilitators for existing treatments, and the characteristics of an “ideal” intervention to address review questions relating to feasibility and acceptability. Furthermore, few primary studies are likely to share the same research question or focus as the planned synthesis. However, this does not mean that these studies may not yield relevant data [34]. “Dropping” the intervention concept makes the search strategy broader for the qualitative component than for its quantitative counterpart. Alternatively, for interventions where context is important (e.g. cultural attitudes or health service specific effects), a qualitative synthesis may implement a narrower interpretation of scope, such as countries with a comparable health system (to facilitate transferability) compared with the SR of clinical trials (which aspires to generalisability). The Cochrane Handbook [6] cautions that seeking to retrieve qualitative studies from a topic-based search strategy designed to identify trials is methodologically unsound. A trial-based search strategy is not designed to identify qualitative studies. Indeed, the trial-based strategy may well achieve a measure of specificity by purposefully excluding many qualitative research types.

Where the scope of quantitative and qualitative reviews is co-terminous a review team can employ a broad approach using subject and topic terms without specifying the study type(s) of interest [8]. Both quantitative and qualitative studies would be identified. However, this method generates large numbers of retrieved records and requires those sifting the abstracts to be equally adept at identifying both types of study. Such an approach is employed at the EPPI-Centre where they routinely conduct reviews of “views studies” alongside reviews of effectiveness [130].

In reviewing the quality of reported search procedures in a sample of published qualitative syntheses, Dixon-Woods et al. observe that search techniques often lack sophistication and are thus likely to miss relevant material [88]. They suggest a need to involve an information specialist in the search process, already well-recognised for quantitative systematic reviews.

### Search strategies and filters

For inclusion in this section, a publication was required to either (i) provide an overview of search strategies and/or methodological filters within the context of QES or (ii) include a substantive discussion of search strategies and/or methodological filters within a published QES or (iii) any mention of search strategies and/or methodological filters within works identified for the “Overviews, summaries and guidance” section. Sixteen items were included in this section [8, 16, 17, 19–25, 34, 37, 44, 77, 88, 90].

The development of pre-specified search strategies using methodological terms has an extensive pedigree within quantitative research, particularly for randomised controlled trials. Methodological “filters” or “hedges” are specially designed search strategies used to retrieve citations of clinically relevant and scientifically sound studies (or reviews) [131]. These search terms are initially suggested by librarians and clinical users, and then, performance metrics are generated for these terms both singly and in combination. The performance of hedges for clinical trials was subsequently enhanced by a Cochrane-associated retrospective indexing initiative. Almost a decade later, the Hedges Project at McMaster University expanded its battery of empirically tested methodological filters to include qualitative research filters for the four principal health-related databases, namely MEDLINE [25], CINAHL [24], PsycINFO [19] and EMBASE [23]. A range of filters (sensitive—to minimise the potential of missing relevant references, specific—to minimise the potential of including irrelevant references and optimal—to determine an efficient trade-off between sensitivity and specificity) is available for each database (Table 5), supported by information about how the filters were developed to help in selecting an appropriate filter (http://hiru.mcmaster.ca/hiru/HIRU_Hedges_home.aspx). A survey of members of the Cochrane Qualitative Methods Network revealed some resistance to the use of filters with associated concerns about whether filters were suitably comprehensive [17]. Notwithstanding these concerns, qualitative filters demonstrate a performance that compares favourably with that for retrieval of trials. More recently, the InterTASC Information Specialists’ Sub-Group (ISSG) of information professionals has produced a Search Filter Resource (www.york.ac.uk/inst/crd/intertasc/) documenting the evidence base for published filters.

**Table 5:** Performance of qualitative filters

Database	Filter type	Filter terms	Sensitivity	Specificity
MEDLINE [PubMed]^a^ [25]	Maximises sensitivity	interview*[Title/Abstract] OR psychology[Subheading:noexp] OR health services administration [MeSH Term]	95	70
	Maximises specificity	Qualitative[Title/Abstract] OR Themes[Title/Abstract]	61	99
	Best balance of sensitivity and specificity	interview*[Title/Abstract] OR interviews[MeSH:noexp] OR experience*[Text Word] OR qualitative[Title/Abstract]	92	92
EMBASE [Ovid, 23]	Maximises sensitivity	interview:.tw. OR qualitative.tw. OR exp health care organisation	94	90
	Maximises specificity	qualitative.tw. OR qualitative study.tw.	57	100
	Best balance of sensitivity and specificity	interview:.tw. OR exp health care organisation OR experiences.tw.	90	90
PsycINFO [Ovid, 19]	Maximises sensitivity	experience:.mp. OR interview:.tw. OR qualitative:.tw.	94	79
	Maximises specificity	qualitative:.tw. OR themes.tw.	50	99
	Best balance of sensitivity and specificity	experiences.tw. OR interview:.tw. OR qualitative.tw.	86	87
CINAHL [Ovid, 24]	Maximises sensitivity	exp study design OR exp attitude OR exp interviews	99	54
	Maximises specificity	exp study design OR exp attitude grounded theory.sh. OR thematic analysis.mp	53	100
	Best balance of sensitivity and specificity	interview.tw. OR audiorecording.sh. OR qualitative stud$.mp.	94	94

^a^Predates introduction of MeSH term qualitative research in 2002

The performance of filters is liable to change over time [22, 125], with new terms being added by the user community, changes to indexing terminology and journal coverage and the appearance of specialist qualitative journals. The utility of the MEDLINE empirically tested filter [25] was compromised, albeit to a minor degree, by the appearance of the MeSH term “qualitative research” in 2002, subsequent to creating the test and validation sets. Differences exist in the indexing of qualitative research within electronic databases such as MEDLINE, EMBASE, PsycINFO and CINAHL [77, 88]. Reviewers should avoid running a filter devised on one database against another database or, less obviously, translating terms on a one-by-one basis from one database to another. In the context of research syntheses, more generally, Cooper [132] encourages searching multiple databases simultaneously where possible, to avoid excessive duplication. However, a simultaneous searching of multiple databases requires that the searcher develop a strategy that is not overly reliant on specific indexing terms, particularly as they might be artificially inverted (e.g. education, professional) and thus retrieve zero hits on databases other than their parent source.

Optimally, a sensitive strategy retrieves individual terms regardless of how they occur within indexing languages although in the example of “Professional AND Education” the numbers of retrieved results and the high proportion of “false hits” would prove prohibitive. It may be useful to undertake a content analysis of the subject headings assigned in each database to determine why indexing terms diverge across databases [22]. The more varied the databases, the more challenging creation of a “standard” search filter for application to all databases becomes, suggesting that search strings need to be “adapted to the idiosyncrasies of each synthesis to achieve the best results” [37]. Generally, methodological filters for qualitative research have undergone little replication and validation [16, 34, 88]. It is not known whether search filters, as developed for health care [20, 23–25], are equally feasible and useful within disciplines such as social work or education.

In the context of filters in general, Jenkins [106] attempts to identify different generations of search filters with corresponding degrees of rigour. Foremost among these are filters developed from a single “gold standard” set of unequivocally relevant references (i.e. identifiable qualitative research studies) and then randomly splitting these records into a development and validation set. Unfortunately, few available filters or hedges for qualitative research meet this empirical standard.

A search strategy to be effective requires (i) that it retrieves relevant records, (ii) that it does not retrieve irrelevant references and (iii) that the collective terms be parsimonious, thereby avoiding redundancy. The third requirement is a particular current concern for the qualitative searching community. Lengthy filters have been devised, collecting together research methodology terms or all possible terms to capture “patient views.” However, once a particular record is retrieved by one term, there is little value in retrieving it again using subsequent terms. A reviewer is primarily interested in retrieving additional different records or in retrieving a record on a subsequent database if it has been missed because of the indexing particularities of a previously searched database [77]. Two case studies [16, 44] suggest that a parsimonious strategy involving the terms qualitative, findings and interview* (as both text word and index term) may perform acceptably well when conducting a search for qualitative research across a range of databases. Such a broad free-text strategy performed particularly well on the CINAHL database [8]. It is critical that this finding is tested across multiple topics and time spans.

Searching with “broad terms” such as “qualitative research,” “qualitative studies” and “interview,” together with their topic terms, may be equally applicable for all databases [90]. In contrast, specific MeSH terms or methodological index terms that aid the identification of qualitative research (“exploratory,” “grounded theory,” “content analysis,” “focus groups” and “ethnography”) provided no additional results and delivered numerous false hits. An evaluation by Shaw et al. [21] confirms that an optimal strategy for retrieving qualitative research will combine specific free-text terms, broad terms and thesaurus terms; relying on one strategy alone would fail to identify relevant records. This evaluation identified low precision for all three types of strategy, with only 4 % of papers proving to be relevant at full-text screening. Campbell et al. [37] describe using the single term “qualitative” for title searches of the ZETOC contents database. Anecdotally, searches of Google Scholar enjoyed some success by adapting the MEDLINE-based “qualitative OR findings OR interview*” strategy, either combined with subject terms or within the “Search within Cited Articles” feature for already-identified relevant citations.

### Supplementary strategies

To be included, a publication was required to either (i) provide an overview of supplementary (i.e. non-topic-based) search strategies within the context of QES or (ii) include a substantive discussion of supplementary (i.e. non-topic-based) search strategies within a published QES or (iii) include any mention of supplementary search strategies within works identified for the “Overviews, summaries and guidance” section. A total of 24 items was thus included in this section [8, 10, 14, 20, 30, 34, 37, 39, 42, 45, 62, 63, 84, 94, 107–116].

Guidance on meta-ethnography advocates that, in order to minimise the risk of missing studies, searchers conduct supplementary searches alongside topic-based database searching [37]. The authors propose a multipronged approach; hand-searching relevant journals; contacting experts in the field of enquiry for curricula vitae and information; and examination of the “grey literature,” conference proceedings etcetera [37]. They rightly advise that decisions should be made at each stage depending on the resources available. Search strategies for qualitative research should extend beyond electronic databases [20, 30, 63] but knowledge about which strategies to use for particular topics is not forthcoming [109]. Several commentators report that supplementary search strategies are useful in compensating for deficiencies of indexing terms and the limited value of “protocol-driven search strategies” [8, 10, 14, 62]. Key journals are hand-searched in case electronic searches are not sufficiently sensitive or where indexers have not assigned adequate keywords [37].

Noticeable is a trend to favour creative approaches to retrieval—most notably Bates’ berry picking approach [107]. Barroso et al. [10] used berry picking as a framework for their search techniques for a meta-synthesis project on women with HIV infection. Combining formal search strategy methods with berry picking may help to expand searching from a broad topic towards “new, unpredictable ideas and directions” and even reformulation of the original query [108]. More recently, berry picking has been revisited, as an alternative to extensive keyword-based approaches [15, 40].

Booth et al. [110] have designed a search approach that seeks to place Bates’ berry picking [107] on a more systematic footing. The CLUSTER approach seeks to maximise identification of associated or linked studies, identifying not only studies that are instrumentally linked as “sibling studies” but also studies that are theoretically or conceptually associated, “kinship studies” [110]. From a single “key pearl citation,” the authors conduct searches to find contextually or theoretically proximate documents. They follow up Citations, trace Lead authors, identify Unpublished materials, search Google Scholar, track Theories, undertake ancestry searching for Early examples and follow up Related projects (embodied in the CLUSTER mnemonic) [110].

Increasingly, reviews of complex interventions require that a review team identifies the conceptual underpinnings and explores the contextual detail. The CLUSTER method aims to retrieve both concepts and contexts [110]. While not all commentators acknowledge that theory can be retrieved in a systematic way, Booth and Carroll [9] have recently devised a structured procedure, BeHEMoTh, of steps to retrieve papers reporting theory.

Dixon-Woods et al. [84] reported that the most common supplementary strategies used alongside bibliographic databases were following up reference lists and hand searching. Subsequently, Hannes et al. [39] found that reference or citation searching was used in more than half the QES in their sample. Other popular search strategies included hand-searching journals, contacting experts or authors or web searching. Reviewers also mentioned personal correspondence, related paper options in existing databases, email discussion lists, footnote chasing, or searching conference abstracts, etc. Other approaches include scanning conference proceedings, contacting professional bodies, searching for grey literature and looking at included studies of earlier reviews, personal correspondence, related paper options in existing databases, email discussion lists, footnote chasing or searching conference abstracts [39, 42].

Greenhalgh and Peacock, frequently cited in support of deficiencies of topic-based search strategies, report an audit of sources for a review of complex interventions, of which a proportion relates to qualitative evidence [62]. Only 30 % of included studies were identified from databases and hand searches. About half of studies were identified by “snowballing” (e.g. reference, footnote and citation tracking) and another 24 % by personal knowledge or personal contact. However, the team had recognised a priori that their topic area was diffuse and ill-suited to keyword-based strategies (in essence becoming self-fulfilling) and report relative percentages where increased effort aimed at one source, e.g. personal contact, makes the remaining sources appear less useful. A rigorous evaluation would study whether items *could* have been identified using databases, regardless of how they *were actually identified*. In another case study, citation searching, reference checking and contact with experts yielded 11 of 41 included studies [20]. The use of citation pearl growing (i.e. using known relevant items to identify supplementary search terms) was of limited value because none of the 10 candidate databases, from which the other 31 included studies were derived, included more than four relevant items and therefore did not offer sufficient data for analysis.

#### Reference checking

Gomersall et al. [115] report that using reference lists of relevant literature identified 38 relevant articles. Critical, however, is how many included studies were identified *uniquely* from reference lists. Similarly, Steen et al. [116] found that backchaining (i.e. checking of reference lists from included studies) identified a further six studies of potential relevance. However, their paper is unclear on how many of these were uniquely identified and subsequently included in the review. Malpass et al. [114] checked reference lists and contacted authors unearthing one relevant paper which was of sufficient quality to be included in the synthesis. Checking the context of a citation within the paper, not just its appearance in a reference list, is particularly helpful when titles are not informative [34]. There is a compelling argument to suggest that checking of references in the full-text of already included, or indeed potentially includable retrieved studies, should not be regarded as a supplementary technique but rather simply as standard good practice.

#### Hand searching

Several reports rate hand searching of relevant journals as “useful” [109]. However, such anecdotes usually lack data on yields or time spent hand searching. Hand searching is particularly indicated where relevant data is “buried” within the text of a paper and the study is not retrieved through electronic searches [34]. Typically, hand searching is a misnomer as browsing of titles and abstracts is facilitated online or, where available, a journal’s search facility offers full-text searching over and above the title and abstract facility offered by most bibliographic databases. Additional time should be allowed for supplementary activities [34]. In published audits, *Qualitative Health Research* [84] and *Journal of Advanced Nursing* [39] were the most common outlets for QES. For meta-ethnographies, France et al. [42] reported that the majority (41 %) were published in nursing or midwifery journals, a higher proportion than identified in an earlier audit (32 %) [39]. Such data may however be confounded by the reported superiority of CINAHL when indexing qualitative publications.

#### Citation checking

Citation checking harnesses the degree of “relatedness” between an original source and its citing paper. However, “related” items may share a topic, methodology or some tangential or obscure connection. Citation checking (forward chaining) may variously and unpredictably perform better or worse when compared to a keyword-based subject search. Atkins et al. [45] found citation searching of limited use locating only three of 44 included studies through this method and consulting with experts combined. Even though some review teams report limited success from citation checking, they may differ in their thoroughness or the extent that they use complementary search strategies and so citation checking should not be ruled out.

#### Contact with authors/experts

The use of experts as a source of potentially relevant citations has received mixed verdicts, from being vital [62, 63] through to simply useful [111–114]. Campbell et al. [37] describe the “striking” importance of consultation with experts, alongside hand searching. As mentioned above, Atkins et al. [45] found consulting with experts of limited use. Again, the performance of supplementary strategies is relative and depends upon what they are compared with. Greenhalgh and Peacock [62] reported that contacts with experts yielded significant suggestions of potentially relevant reports when reviewing service-level innovations in health care organisations. Pearson et al. reported that contacting authors of included reports was not an effective use of time or resources. Contact with authors yielded 13 potential leads of which only one poor-quality report was included [109]. In part, these differences may be topic-specific, but they may equally reflect how good a review team’s networks are and thus represent a source of potential bias [109].

#### Other methods

Pearson et al. [109] identify a further targeted search strategy using programme names for particular initiatives. This supplementary technique is one component of the CLUSTER methodology [110]. Pearson et al. [109] conclude that it is unclear from published reports why certain topics or supplementary approaches yield more positive results than others. Rather than endorsing one approach over another, this evidence illustrates the challenges of searching across topics that are poorly defined by database keywords. Retrospective analyses of different search approaches require more detailed reporting than presently available [94]. For the moment, a review team must judge how they should allocate their overall search resource between topic-based searching and other approaches for their specific topic. However, a multiple search strategy is more likely to identify relevant qualitative research than one relying solely on electronic searching [37].

### Standards

For inclusion, a publication should either (i) provide a standard for reporting literature searches within the context of QES or (ii) include standards for reporting of literature searching within a wider reporting standard or (iii) any mention of reporting or documentation of search strategies for QES within works identified for the “Overviews, summaries and guidance” section. Seventeen items were included in this section [6, 8, 12, 14, 15, 21, 39, 42, 45, 46, 54, 70, 84, 88, 89, 101, 117].

There remains a high degree of consensus that QES should be systematic, explicit and reproducible [8, 54, 89]. Weed [70], creator of meta-interpretation, observes that the audit trail serves not to enable “member checking” but to make the search transparent and “demonstrate the ‘reasonableness’ of the analysis.” Weed [70] subsequently suggests a level of detail in QES reports that should include the extent of theoretical sampling and how and why and on what basis studies have been chosen for inclusion in each iteration. He further advocates that complete reporting should include processes by which studies are subsequently excluded, reasons for their exclusion; an interim analysis at the end of each iteration and processes by which concepts for further theoretical sampling has been identified.

Audits of reports of published qualitative evidence syntheses reveal disappointingly low standards of reporting of search processes [12, 39, 84]. Typically, neither the search strategy nor the databases searched are detailed in the published report [84]. Similar limitations in reporting of search strategies were observed in a recent survey of meta-ethnographies [42]. Both CRD [8] and Cochrane [6] champion the importance of reporting standards for search methods, including documenting the methods for sampling. They highlight proposed Standards for Reporting Literature searches (STARLITE) as a useful resource [12].

Reporting of systematic reviews is prescribed by the PRISMA, formerly QUOROM, statement. In an attempt to mirror this approach within QES, an international collaboration has produced a tentative reporting standard. The ENhancing Transparency in REporting the synthesis of Qualitative research (ENTREQ) draft statement [88] recommends the use of the PRISMA flowchart [128] for reporting, searching, screening and identifying studies for inclusion in the QES. ENTREQ is influenced by STARLITE [12] in recognising the need to specify the sampling strategy (item 3)—a feature not typically included when searches are comprehensive by default. Table 6 presents the four items from ENTREQ that specifically relate to literature searching and maps these to the elements of the STARLITE mnemonic. However, uptake of ENTREQ [88] is low. A recent review of meta-ethnography reporting [42] observed that only one of 19 papers published since ENTREQ’s publication had used the proposed standard to guide its reporting.

**Table 6:** ENTREQ items relating to literature searching

ENTREQ item [88]	Approach	STARLITE [12]
3 approach to searching	Indicate whether search was pre-planned or iterative; using comprehensive or theoretical sampling	S—sampling strategy
4 inclusion criteria	Specify inclusion/exclusion criteria (e.g. in terms of population, language, year limits, type of publication, study type)	T—type of studies R—range of years L—limits I—inclusions/exclusions
5 data sources	Describe information sources used (e.g. electronic databases)	E—electronic sources A—approaches
6 electronic search strategy	Describe literature search (e.g. provide electronic search strategies and search limits)	T—terms used

Several authors have identified a corresponding need to improve the quality of reporting of primary qualitative research, many focusing on the utility of structured abstracts [45]. Although this review emphasises potential differences with searching for an SR of clinical trials, both types of review benefit from attempts to improve reporting of search methods. Thus, Kable et al. [117] provide a 12-step general strategy for documenting the search process for a literature review, heavily informed by qualitative work [12, 14, 21, 101]. Niederstadt and Droste [133] specify requirements for reporting and presenting information retrieval processes for health technology assessment, and these too inform presentation of search results for a QES.

Finally, we remark upon a paradox, now recognised in the QES community, that iterative approaches using innovative, yet appropriate, sampling techniques may reflect more informed sophisticated and topic-specific approaches to searching and yet be correspondingly more difficult to report. Reviewers face a choice between a simple, yet easily reported, strategy and a complex, “messy” but accurate, strategy that is more difficult to describe and present. Reviewers should make literature search processes as transparent as possible, even when complex [15]. However, little practical guidance exists on how to achieve such transparency. France et al. [42] speculate that one reason why comprehensive searches persist may be attributed to the dominance of established methods and guidance for conducting and reporting quantitative reviews of trials [8, 128, 129]. Toye and colleagues [46] reflect on how the shaping influence of the Cochrane Collaboration impacted on their decisions to conduct and report their QES and thus satisfy external expectations for rigour.

## Discussion

While there appears to be considerable consensus in relation to the methodology of searching for qualitative research, the findings from this methodological overview should be treated with caution. A limited number of authors and teams are particularly influential within this narrow, specialist area of information retrieval. Furthermore, much accepted opinion can be attributed to the fact that different commentators are drawing upon and citing the same limited set of references in support of their opinions. The methodological guidance is particularly derivative from a few key influential works. Furthermore, retrospective analyses limit the extent to which investigators can take account of search strategy design, database interface and accuracy of database indexing [94]. Future analyses should collect data on search strategy design, database interface and indexing prospectively to allow review teams to consider the impact of these factors on overall search performance. Finally, the review reveals a paucity of empirical data. Much guidance is based on personal or organisational experience, limited case studies or overworked, and occasionally misapplied, empirical studies, for example the implication that the specific emphasis of search approaches appropriate to a meta-narrative on diffusion of innovations is generically transferable to all QES topics [62]. Table 7 tentatively suggests some starting principles, with reference to the 7S sections of this review, to inform guidance irrespective of the review producer. On a positive note, we can detect increasing transparency in search methods. Hannes et al. [3] compared published data from 2005 to 2008 [39] with data from 1988 to 2004 [84]. Considerably, more QES papers described the databases they had searched, more reported supplementary search strategies and more chose to specify their search terms.

**Table 7:** Some starting principles for qualitative searching

Component	Starting principles
Sampling	Where approaches other than comprehensive sampling are used, reviewers must justify their sampling strategy, match it to their synthesis method and describe fully how it was implemented.
Sources	For health topics, MEDLINE and CINAHL are considered a minimum, augmented by topic-specific and setting-specific sources. Reviewers should devise specific strategies to find specific types of grey literature, where included.
Structured questions	In the absence of empirical data on effectiveness of structured approaches, the question structure should be selected to match the purpose and focus on the review. When accompanying a review of clinical trials, the two review questions may or may not be co-terminous.
Search procedures	Given the comparatively low yield of qualitative topic-based searches, reviewers should privilege specificity (retrieval of relevant items). Retrieved relevant items can then be used as a starting point for developing supplementary search techniques. Reviewers should compensate for reported deficiencies in indexing by using a broad range of supplementary strategies.
Search strategies and filters	Filters should be commensurate with the intended purpose of the review. When extensive supplementary strategies are being employed to offer improved sensitivity, the topic-based searches may use a simple filter (using terms such as qualitative OR findings OR interview).
Supplementary strategies	Reference checking must be a default for every review. For diffuse topics, or those with significant variation in terminology, hand searching, citation searching or contact with authors/experts may be relatively productive. Where context or theory is particularly important, the CLUSTER method [110] may be appropriate. Trial identifiers (ISRCTN or trial name) may be useful for sibling or kinship studies for trials.
Standards	In the absence of a consensual standard for reporting, ENTREQ [88], supplemented by PRISMA [128] and STARLITE [12] where necessary, should be used when reporting a search.

### Towards a research agenda

McGinn et al. [91] recommend that review teams partner with librarians or information specialists to share the outcomes of case studies that showcase thorough searches and examine their yield. There is a particular need to report data, either within reviews themselves or in subsequent retrospective methodological studies, on where included studies could have been found as well as how they actually were found. The caveat is that reviewers report considerable differences in yields from different sources for different topics (e.g. even for two meta-ethnography case studies by the same team [37]). Consequently, review teams cannot predict whether topics are more likely to be similar or different, with what has worked previously not being a guarantor of subsequent success in a different topic [37]. Indeed, the degree to which past performance is a predictor of future performance is currently unknown [94].

A review of the methodological guidance reveals a need to balance development of generic guidance with development of guidance specific to particular methods of synthesis. The development of specific guidance, where methods of sampling, searching and synthesis are all aligned, is a potential route for reconciling the comprehensive versus purposive sampling debate. Important developments for QES reporting standards are the National Institute for Health Research funded Realist And Meta-narrative Evidence Syntheses: Evolving Standards (RAMESES) project for meta-narrative [118] and realist reviews [119] and the Meta-Ethnography Reporting Guideline (eMERGe) project for meta-ethnographies [120].

Table 8 compiles a research agenda with reference to the “7S” framework of this review. This review possesses several limitations. For inclusion, references must include terms specifically related to searching or retrieval in their titles or abstracts, or cite a limited number of key texts, or be referred to from previously identified items. It is increasingly prohibitive to examine the full-text of all papers reporting QES. Individual reviews may explore innovative methods of information retrieval but not showcase their methodology. However, it is unlikely that this review has completely overlooked important issues given the extent of included articles, studies and guidance. While patterns of co-citation or theoretical saturation are largely unexplored within a methodological context, the reviewer reached a point where no additional items were being identified. Some key items were purposely excluded because they did not differentiate between quantitative and qualitative searching approaches. However, these might be useful, particularly where a mixed methods review is being conducted [134–138].

**Table 8:** Towards a research agenda

Component	Research priorities
Sampling	Comparison of yields from exhaustive versus comprehensive sampling [32]. Informed matching of sampling to search methods to synthesis approaches
Sources	Audits of relative yield [77]
Structured questions	Exploration of techniques for automated document clustering to provide initial overview of available evidence across a broad range of topic areas [140, 141]
Search procedures	More empirical testing of different approaches to searching [142, 143]. Exploration of iterative and theory-based approaches [41]
Search strategies and filters	Ongoing rigorous development of methodological filters comparing parsimonious and exhaustive lists. Filters for different qualitative study types [34], process evaluations and mixed methods studies [21, 44]. Search strategies by discipline (e.g. social work), by application (e.g. patient satisfaction) or for theories
Supplementary strategies	Audits and evaluations of relative yield [16]
Standards	Development of consensual reporting standards for QES iterative search approaches; audits of reporting standards generally and for specific methods; standards to handle [39, 84]

With regard to the quality of the identified papers, this review detected a high proportion of overviews, occasionally based on or supported by one or more case studies. Indeed, case studies were the most prevalent method used to advance observations on search methodology, with the corresponding weakness that lessons from individual case studies may not be transferable. The shortage of comparative designs or validation studies is likely to be indicative of a corresponding dearth of funded projects exploring methodological aspects of searching for qualitative studies. Similarly, outside the case study evidence base, wider insights derive either from opportunistic samples or from analysing convenience samples of published QES. Notwithstanding the fact that many guidance documents exist in this domain, these too are limited by the weak quality of the evidence used to underpin published recommendations.

## Conclusions

QES is an exciting and rapidly developing methodological field, evidenced by a proliferation of methods and of published examples. Decisions regarding search strategy and screening hinge upon such considerations as the review aims, resources, availability of studies and epistemological viewpoint [46]. However, the popularity of QES should not mask the poor empirical base that exists for many decisions within the searching process. Methodological overviews are largely populated by common empirical studies which are frequently referenced as authoritative. As with quantitative reviews, there is little empirical data to support the merits of question formulation [101]. Yields from particular databases appear to be largely review specific. Empirical research is required to examine suggestions in the literature that thorough searching of a small number of databases [16, 22, 90], supplemented by other searching methods, may be more efficient than searching across a wider range of databases. We are beginning to learn the merits of different sampling approaches and their alignment to named qualitative synthesis methodologies [38]. Limited but important evidence exists to suggest that a few qualitative methodology keywords may perform equally well to more extensive filter terms [8, 16, 17, 44]. Strategies for retrieving books and theses need to be specified with specific agendas remaining to be advanced in terms of searching for process evaluations or mixed-methods studies. Finally, progress has been made in reporting QES, but these standards have neither been validated in the appropriate community nor extended to cover a broad range of QES methodologies [88]. The QES search methodology research agenda remains ripe for harvesting.

## Appendix

((("meta ethnography" OR "meta ethnographic") OR ("meta synthesis") OR (synthesis AND ("qualitative literature" OR "qualitative research")) OR ("critical interpretive synthesis") OR ("systematic review" AND ("qualitative research" OR "qualitative literature" OR "qualitative studies")) OR ("thematic synthesis" OR "framework synthesis") OR ("realist review" OR "realist synthesis") OR ((("qualitative systematic review" OR "qualitative evidence synthesis")) OR ("qualitative systematic reviews" OR "qualitative evidence syntheses")) OR (("quality assessment" OR "critical appraisal") AND ("qualitative research" OR "qualitative literature" OR "qualitative studies")) OR (("literature search" OR "literature searching" OR "literature searches") AND ("qualitative research" OR "qualitative literature" OR "qualitative studies")) OR (Noblit AND Hare)) OR ("meta narrative" OR "meta narratives" OR "narrative synthesis") OR ("realist reviews" OR "meta study" OR "meta method" OR "meta triangulation")) OR (CERQUAL OR CONQUAL).
